# Supplementary material for: Photonic Label-Free Biosensors for Fast and Multiplex Detection of Swine Viral Diseases
Source: Sensors (Basel). 2022 Jan 18;22(3):708. doi: 10.3390/s22030708 (PMC8838678; doi:10.3390/s22030708)
Supplement: Supplementary file 1 [file sensors-22-00708-s001.zip › sensors-1526624-supplementary.pdf]

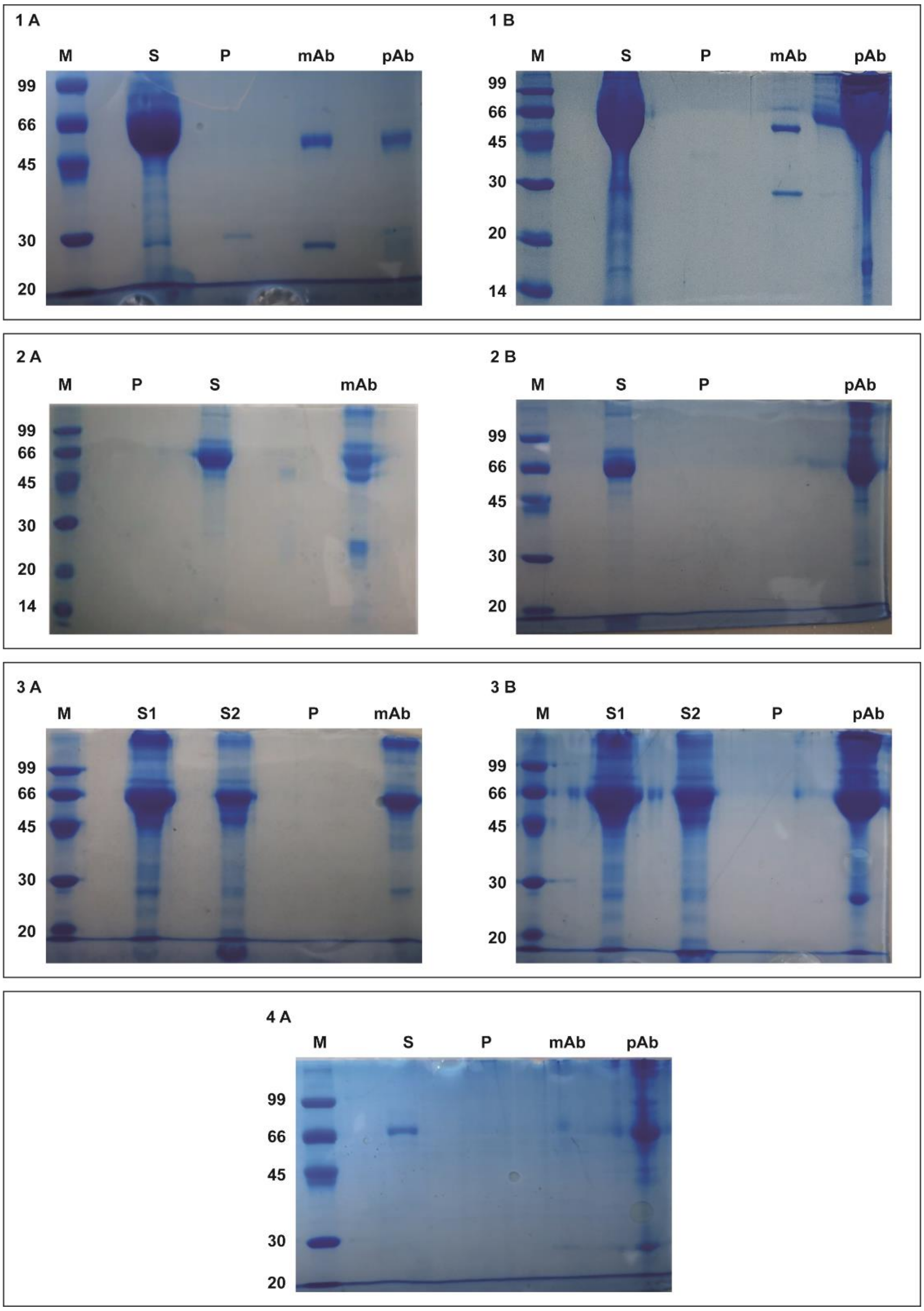

Figure S1. Panel 1A: SDS-PAGE electrophoresis of the PCV2 virus sample (line S), recombinant ORF2 capsid protein (line P) and purified mAb and pAb anti-PCV2 (lines mAb and pAb). **Panel 1B:** SDS-PAGE electrophoresis of the CSFV virus sample (line S), recombinant E2 envelope protein (line P) and purified mAb and pAb anti-CSFV (lines mAb and pAb). **Panel 2A and 2B:** SDS-PAGE electrophoresis of the PRRSV virus sample (line S), recombinant NP nucleocapsid protein (line P) and purified mAb and pAb anti-PRRSV (lines mAb and pAb). **Panel 3A and 3B:** SDS-PAGE electrophoresis of the PPV ST and SK-6 virus samples (lines S1 an S2), recombinant VP2 protein (line P) and purified mAb and pAb anti-PPV (lines mAb and pAb).**Panel 4A:** SDS-PAGE electrophoresis of the ASFV virus sample (line S), recombinant p30 capsid protein (line P) and purified mAb and pAb anti-ASFV (lines mAb and pAb).

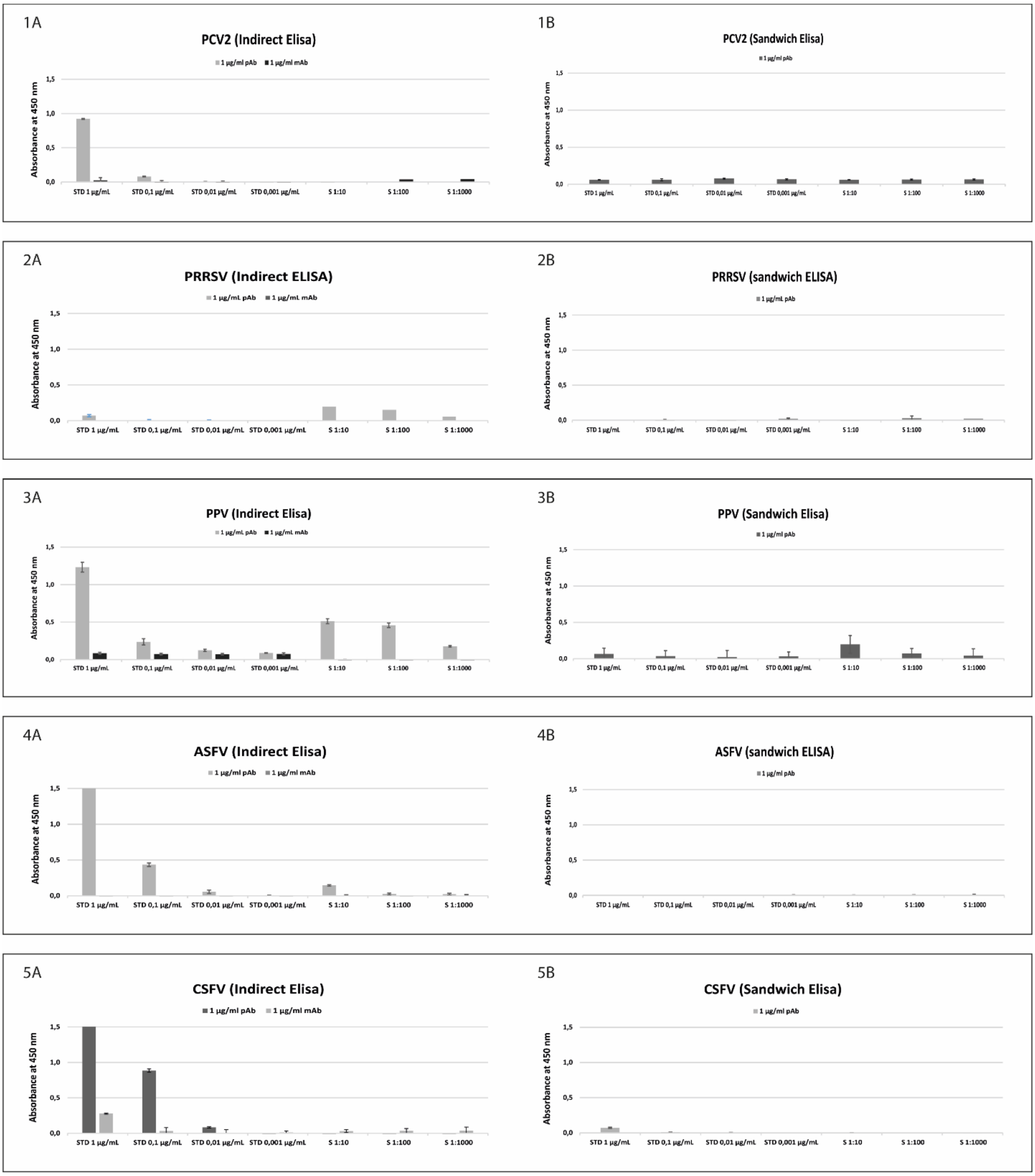

Figure S2. Panel 1A: Indirect ELISA of the pAb and mAb against the PCV2 virus sample (S) and the recombinant ORF2 capsid protein (STD). **Panel 1B:** Sandwich ELISA of the mAb (capture element) and pAb (detection element) against PCV2 virus sample (S) and the recombinant ORF2 capsid protein (STD). **Panel 2A** Indirect ELISA of the pAb and mAb against the PRRSV virus sample (S) and the recombinant NP nucleocapsid protein (STD). **Panel 2B:** Sandwich ELISA of the mAb (capture element) and pAb (detection element) against the PRRSV virus sample (S) and recombinant NP nucleocapsid protein (STD). **Panel 3A** Indirect ELISA of the pAb and mAb against the PPV virus sample (S) and the recombinant VP2 protein (STD). **Panel 3B:** Sandwich ELISA of the mAb (capture element) and pAb (detection element) against the PPV virus sample (S) and recombinant VP2protein (STD). **Panel 4A:** Indirect ELISA of the pAb and mAb against the ASFV virus sample (S) and the recombinant p30 capsid protein (STD). **Panel 4B:** Sandwich ELISA

of the mAb (capture element) and pAb (detection element) against the ASFV virus sample (S) and the recombinant p30 capsid protein (STD). **Panel 5A:** Indirect ELISA of the pAb and mAb against the CSFV virus sample (S) and the recombinant E2 envelope protein (STD). **Panel 5B:** Sandwich ELISA of the mAb (capture element) and pAb (detection element) against the CSFV virus sample (S) and the recombinant E2 envelope protein (STD).
